# COMMENTARY ON “THE EFFECTS OF MODERATE-INTENSITY AEROBIC EXERCISE ON COGNITIVE FUNCTION IN INDIVIDUALS WITH STROKE-INDUCED MILD COGNITIVE IMPAIRMENT: A RANDOMIZED CONTROLLED PILOT STUDY”

**DOI:** 10.2340/jrm.v57.42555

**Published:** 2025-02-25

**Authors:** Simran Sunil KHUTARKAR, Divya KHATI, Sidharth BANSAL

**Affiliations:** 1 Department of Physiotherapy, School of Allied Medical Sciences, Lovely Professional University, Jalandhar, Punjab, India; 2 Department of Physiotherapy, Rayat-Bahra University, Chandigarh-Ropar, Greater Mohali, Punjab, India

*To the Editor*,

I would like to begin by expressing my appreciation for the article by Huang et al. (1). The authors have made a valuable contribution to stroke rehabilitation by focusing on cognitive recovery, an often underexplored area in stroke research. With its randomized controlled design, this study provides crucial insights into moderate-intensity aerobic exercise’s effects on cognitive function – particularly in working memory, visuospatial/executive functions, and delayed recall – in individuals with stroke-induced mild cognitive impairment (MCI). The treatment strategy is highly reproducible, allowing for potential implementation in diverse rehabilitation settings. Additionally, the detailed interpretation provided concerning the outcome measures such as the Montreal Cognitive Assessment (MOCA) and Mini-Mental State Examination (MMSE) add credibility to the findings, and the results are promising. The attention to both standard rehabilitation therapy and the addition of aerobic exercise as an adjunctive therapy reflects an innovative approach that could significantly influence future rehabilitation protocols for stroke patients.

However, as with any scientific endeavour, several areas could be improved to enhance the rigour and overall contribution of the study to the field. Critical evaluation of certain aspects of the methodology, statistical analysis, and reporting would improve the study’s clarity and offer more definitive conclusions. Below, I outline several points of critique that could strengthen the study, beginning with a foundational issue in research design: hypothesis formulation.

A well-structured scientific study should present both a null and an alternative hypothesis to guide the statistical analysis and interpretation of results (2), which was found to be lacking in the current study. The null hypothesis could propose that moderate-intensity aerobic exercise may not affect cognitive function in stroke-induced MCI patients, while the alternative hypothesis would suggest that such exercise may positively impact cognitive function. The absence of specified hypotheses limits the interpretive framework of the study and leaves the reader without a clear understanding of the research question’s foundational structure. This issue could be addressed by clearly articulating the hypotheses in the introduction or methods section, making the study more methodologically sound and scientifically transparent.

Additionally, the study does not specify the area of haemorrhage or ischaemia that caused the stroke in participants. Anatomical location is crucial in understanding potential cognitive outcomes, as different brain regions are associated with distinct cognitive functions (3). For instance, strokes affecting the frontal lobe may impair executive functions, whereas strokes in the temporal lobe could affect memory. It becomes difficult to draw meaningful conclusions regarding the intervention’s efficacy in improving specific cognitive functions like working memory or visuospatial skills without clarifying the brain areas affected in each participant. Including this information would enhance the study’s clinical relevance by correlating brain damage areas with cognitive outcomes, thus providing a clearer rationale for choosing outcome measures like working memory and visuospatial function.

The wide time range post-stroke, as noted in the abstract (2–12 months), introduces variability in recovery stages, potentially confounding the study’s results. Stroke recovery is a dynamic process, and cognitive improvements in the early stages (within the first 3 months post-stroke) might occur naturally as part of spontaneous recovery, while improvements in the chronic phase (after 6 months) are more likely due to therapeutic intervention (4). Grouping participants who are as early as 2 months post-stroke with those who are 12 months post-stroke may obscure the true effects of aerobic exercise. The study could benefit from either narrowing this time window or conducting subgroup analyses to explore whether the timing of the intervention influences its effectiveness. This would provide more detailed data on how aerobic exercise affects cognitive function at different stages of stroke recovery.

**Fig. 1:**
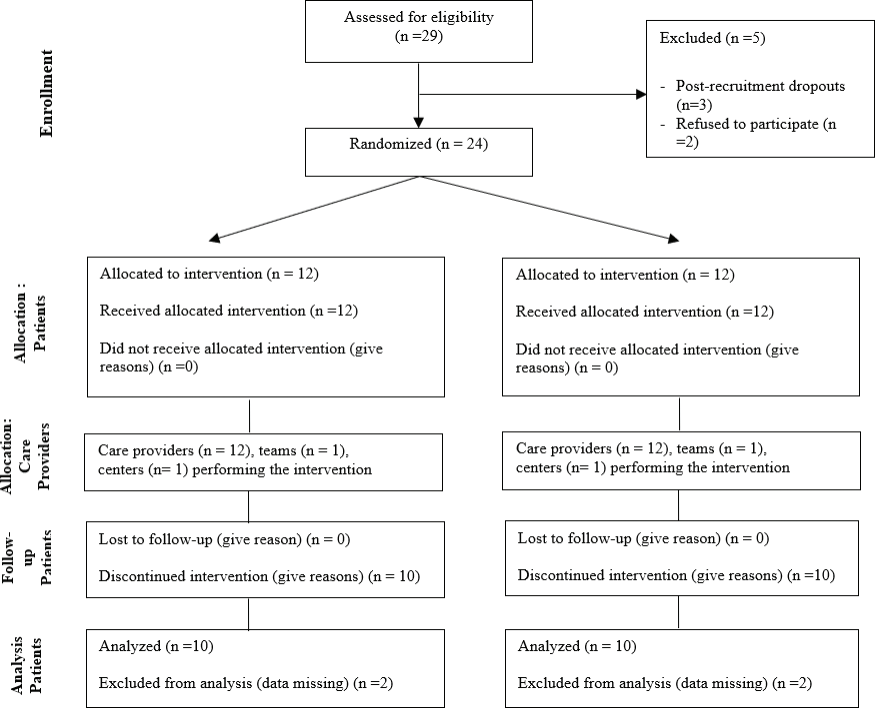
CONSORT flow diagram.

In Fig. 1, which illustrates the study’s flow of participants, the authors do not adhere to the Consolidated Standards of Reporting Trials (CONSORT) guidelines. CONSORT flow diagrams are essential in randomized controlled trials as they provide transparency in reporting participant allocation, intervention adherence, and dropouts (5). A CONSORT-compliant flowchart would improve reporting quality, allowing readers to assess the randomization process, intervention consistency, and result validity. The omission of this standardized reporting tool raises concerns about the trial’s robustness, and I strongly recommend that the authors revise Fig. 1 to comply with CONSORT guidelines to enhance the study’s credibility. The authors could consider a CONSORT diagram (Fig. 1) at the end of the study to enhance clarity and transparency.

The study’s focus on cognitive function could be expanded to provide a more comprehensive analysis. While the authors assess working memory, visuospatial skills, and delayed recall, it would be beneficial to include other cognitive domains such as attention, processing speed, and language, as these are also commonly affected by stroke. Occupational background, an important factor influencing cognitive outcomes, is notably absent. Research, such as the study by Li et al. (2022) on occupational factors in cognitive outcomes post-stroke, demonstrates that cognitively demanding jobs are often associated with better baseline cognitive function and recovery. Including participants’ work profiles would provide context for cognitive function variability and improve the interpretation of results (6). Additionally, the study’s broad age range (34–79 years) introduces variability, as cognitive function naturally declines with age. This, along with age-related differences in cardiopulmonary fitness, may affect exercise responses and cognitive outcomes (7). Stratifying participants by age or controlling for this variable could address these confounders and enhance results reliability.

The authors did not perform a statistical analysis to determine the significance of pre- and post-intervention values. Although the results indicate potential cognitive function improvements in the experimental group, the absence of statistical evidence makes it unclear whether these changes are significant. Utilizing a non-parametric test, such as the Mann–Whitney–Wilcoxon test, could have addressed this gap (8). Reporting *p*-values, confidence intervals, or effect sizes would provide clarity on whether the observed differences are meaningful or simply due to chance. Without this essential data, the study’s conclusions remain speculative, limiting the ability to evaluate the intervention’s actual impact.

Another important consideration is the lack of clarity on monitoring heart rate (HR) and peak oxygen consumption (PVO_2_) during the intervention. These are key physiological markers in aerobic exercise, and without this information it is challenging to ensure that all participants exercised at the same intensity level. This is especially relevant given the broad age range, as older participants likely have different cardiovascular responses to exercise. Reporting how HR and PVO were monitored and controlled would ensure consistency in the exercise intervention across participants.

Furthermore, the authors do not mention participants’ work profiles, which could significantly impact cognitive function. Occupational background has been shown to influence cognitive performance, with cognitively demanding jobs often associated with higher cognitive function in later life. Adam et al. (9), in their article, discuss the influence of work profiles on cognitive reserves. Omitting this information is a significant oversight, as it would help contextualize participants’ baseline cognitive function and their response to the intervention.

The authors mention that a non-parametric test was used for statistical analysis but do not specify which test. Non-parametric tests are appropriate when data do not meet normality assumptions, but specifying the test is essential for interpreting the results. This lack of detail undermines data analysis transparency, and I recommend that the authors clarify the statistical methods used to enhance methodological rigour.

In conclusion, while the study provides promising preliminary evidence on moderate-intensity aerobic exercise’s cognitive benefits in individuals with stroke-induced MCI, several aspects could be refined to improve the study’s rigour and clinical relevance. Addressing issues such as hypothesis formulation, stroke location, and timing post-stroke, adherence to CONSORT guidelines, and statistical transparency would strengthen the study. Additionally, expanding cognitive assessments and detailing participants’ work profiles and physiological responses would offer a more comprehensive understanding of the intervention’s effectiveness. I commend the authors for their work and encourage them to consider these suggestions in future research to further contribute to stroke rehabilitation.

Thank you for the opportunity to share our feedback on this important study.
